# Use of Microbiological and Chemical Data to Evaluate the Effects of Tourism on Water Quality in Karstic Cenotes in Yucatan, Mexico

**DOI:** 10.1007/s00128-023-03761-1

**Published:** 2023-06-23

**Authors:** Flor Arcega-Cabrera, Karina León-Aguirre, Fernando Enseñat-Soberanis, Germán Giácoman-Vallejos, Gabriela Rodríguez-Fuentes, Ismael Oceguera-Vargas, Elizabeth Lamas-Cosío, Nuno Simoes

**Affiliations:** 1 Unidad de Química en Sisal, Facultad de Química, Universidad Nacional Autónoma de México, Puerto de Abrigo Sisal, Yucatán, 97355 México; 2 Facultad de Ciencias Antropológicas, Universidad Autónoma de Yucatán, Km 1 Carr. Mérida-Tizimin, Mérida, Yucatán 97305 México; 3 Facultad de Ingeniería, Universidad Autónoma de Yucatán, Av. Industrias No Contaminantes por Anillo Periférico Norte, Apdo. Postal 150, Mérida, Yucatán México; 4 Facultad de Ciencias, UMDI-Sisal, Universidad Nacional Autónoma de México, Puerto de Abrigo S/N, Sisal, 97356 Mexico; 5 International Chair for Coastal and Marine Studies, Harte Research Institute for Gulf of Mexico Studies, Texas A and M University-Corpus Christi, Corpus Christi, TX 78412 USA; 6 Laboratorio Nacional de Resiliencia Costera (LANRESC), Laboratorios Nacionales, CONACYT, Sisal, 97356 Mexico

**Keywords:** Cenotes, Ecotourism, Water quality, Hydrogeology, Fecal matter

## Abstract

Cenotes are spectacular karst formations in Yucatan, Mexico, often used for recreation. However, their impact on water quality has yet to be explored in detail. Therefore, during Easter, water samples were collected from four cenotes to identify variations in water quality associated with the presence of tourists. PCO of water quality, before (PH) and during Holy Week (HW) in 2019, explained 49.02% of the total variation. The indicators contributing to the first principal coordinate’s variation were Sr, K, sulfate, and chloride (0.89). Whereas, alkalinity, temperature, conductivity, nitrate, and ORP contributed to the second PC. PERMANOVA indicated a significant interaction between “cenote” and “condition” factors, and post hoc paired comparisons indicated significant differences between PH and HW conditions. Significant correlations varied among the four cenotes as the result of hydrogeological differences. Whereas, numbers of visitors were correlated with at least one fecal-matter indicator, demonstrating anthropogenic influence on the cenotes’ water quality.

## Introduction

The Yucatan peninsula is characterized by its karstic nature, a region without rivers or surficial streams and slight topographic elevation (Schmitter-Soto et al. 2002; Herrera-Silveira et al. 2004; Arcega-Cabrera et al. 2014). Thus, groundwater is the only freshwater source in the state (Aranda-Cirerol et al. 2011). This karst aquifer hosts large amounts of groundwater resources that contain precious and highly vulnerable environments such as sinkholes, mangroves, and seagrass (Bauer-Gottwein et al. 2011, Camacho-Cruz et al. 2020, Cejudo et al. 2020). It is one of the world’s unique regions concerning the character and manifestation of karst processes (Lebedeva et al. 2017), since it is dominated by limestones and dolomites with abundant inundated cave systems and geological formations produced by rock dissolution (Scholz et al. 1995).

Particularly, typical features of this system are the cenotes (from the Maya d’zonot), which are collapsed dolines that intersect the water table (Melo Zurita 2019, León‑Borges et al. 2020). According to the Secretaría de Desarrollo Sustentable’s (SDS Yucatan State) database, there are more than 3,000 registered cenotes and caves within the state (Angyal et al. 2020). Although widely dispersed across Yucatan, a dense region of cenotes forms an arc, extending southwest from Celestun to Cuzama and northeast from Cuzama to Dzilam de Bravo, which is called the Ring of Cenotes (RC) (Pérez-Ceballos et al. 2012). Numerous productive and domestic activities take place around the RC, as well as in nearby areas (Arcega-Cabrera et al. 2014).

In the 1980s, Yucatan experienced the takeoff of tourism development (Lebedeva at al 2017). Tourism is one of the activities that many communities in the Maya area have embraced because it provides jobs and income. As part of this, government programs promote the use of cenotes as tourist attractions. As of 2020, 113 cenotes had been certified for tourism-recreational use (SDS 2021, SEFOTUR 2021). Therefore, cenotes, located both in and outside the RC, have become an essential part of the activities offered by communities that depend on eco-tourism in Yucatan. Unfortunately, this growing interest has resulted in some cenotes experiencing an exponential increase in visitors, which may negatively impact water (Enseñat-Soberanis et al. 2020). Thus, water quality for recreational use is of interest since Mexico lacks regulations for recommended values of human exposure to contaminated water, which can generate an environmental and public health problem. Thus, measurements should be taken to promote the sustainable use of cenotes (Hoogesteijn Reul et al. 2015).

Tourism associated with cenotes brings large numbers of visitors, requiring the installation of significant infrastructure, like doors, pathways, lights, roads supporting vehicular traffic, and restrooms, all of which can be sources of contamination. Lights that are used to highlight certain features in caves and help guide people, could cause algal growth which is unsightly and can dissolve the underlying rock. Moreover, visitors may directly impact the cenotes’ ecosystems in several ways. The most immediate and visible is litter, both above and under the water. They can also negatively impact caves through respiration, which increases levels of CO_2_ and potentially leads to the dissolution of speleothems (Onac and van Beynen 2021). As well, some cosmetics contain heavy metals (HMs), which lead to contamination and represent a risk for aquatic life and human health (Ayenimo et al. 2010; Arshad et al. 2020). Inorganic groundwater contaminants, like nitrate and heavy metals, have been detected in groundwater in the Yucatan Peninsula; and, they have been attributed mainly, but not solely, to waste from agricultural activities and animal husbandry (Arcega-Cabrera et al. 2021). Karstic aquifers are known to be particularly vulnerable to anthropogenic contaminants such as nitrate (Visser et al. 2021). Therefore, considerable efforts have been made to promote the reduction of nitrate inputs, as well as enhance attenuation processes in groundwater in some parts of the world (Yang et al. 2020). However, increases in groundwater nutrient loads are generally attributed to domestic wastewater discharges from nearby urban areas. Direct contributions from human excrements have been ignored and remain unknown, although some studies on water quality in cenotes located around tourist zones in the Yucatan Peninsula have reported nutrients (Camacho-Cruz et al. 2019) with ammonium (average values of 0.01 mg L^-1^), nitrate (7.90 mg L^-1^), nitrite (0.01 mg L^-1^), and orthophosphate (0.01 mg L^-1^). Average reported results in the physicochemical parameters of conductivity and total dissolved solids (TDS) are 1410 µS cm^-1^ and 865 mg L^-1^ respectively (Hernández-Mendoza et al. 2022). Additionally, Leal-Bautista et al. (2011) reported total coliform from 4 to 298 CFU 100 mL^-1^. Whereas, Borbolla-Vazquez et al. (2020) reported total coliforms from 3 to 240 CFU 100 mL^-1^ and fecal coliforms above 2400 CFU 100 mL^-1^.

Water-based tourism, including tourism at cenotes, has increased the presence of contaminants in groundwater systems (Nava-Galindo 2015; Arshad et al. 2020; Silva and Mattos 2020, Casas-Beltrán et al. 2021). Besides, ill-constructed sanitary services may release their contents to the cenotes (Hoogesteijn Reul et al. 2015, Arcega-Cabrera et al. 2021).

However, the solution is not to forbid people from swimming in the cenotes but to apply measures that control and minimize this contamination (Enseñat-Soberanis 2020). Since there is no sanitation infrastructure adapted to the karst system in the tourist-recreational cenotes in Yucatan, the preservation of water quality depends on regulation strategies, monitoring programs, and site-specific management.

Therefore, in this study, we examine water quality parameters, including pH, dissolved oxygen (OD), electric conductivity (EC), alkalinity, nitrate (NO_3_^-^), nitrite (NO_2_^-^), ammonium (NH_4_^+^), orthophosphate (PO_4_^=^), chlorides, sulfates, copper (Cu), cadmium (Cd), chromium (Cr), arsenic (As), total coliforms, fecal coliforms, and *Enterococcus*. These parameters were measured in water samples collected from four eco-tourist cenotes. Sampling was conducted two weeks before the Easter vacation and over a day-long period in each sampled cenote during Easter vacation in 2019. Easter was chosen because it is the period with the highest influx of visitors. The results from these analyses were used to evaluate the response of water quality variables between the pre-tourist season and the high season (Easter vacation) to document probable water quality perturbations related to this activity.

## Materials and Methods

**Fig. 1:**
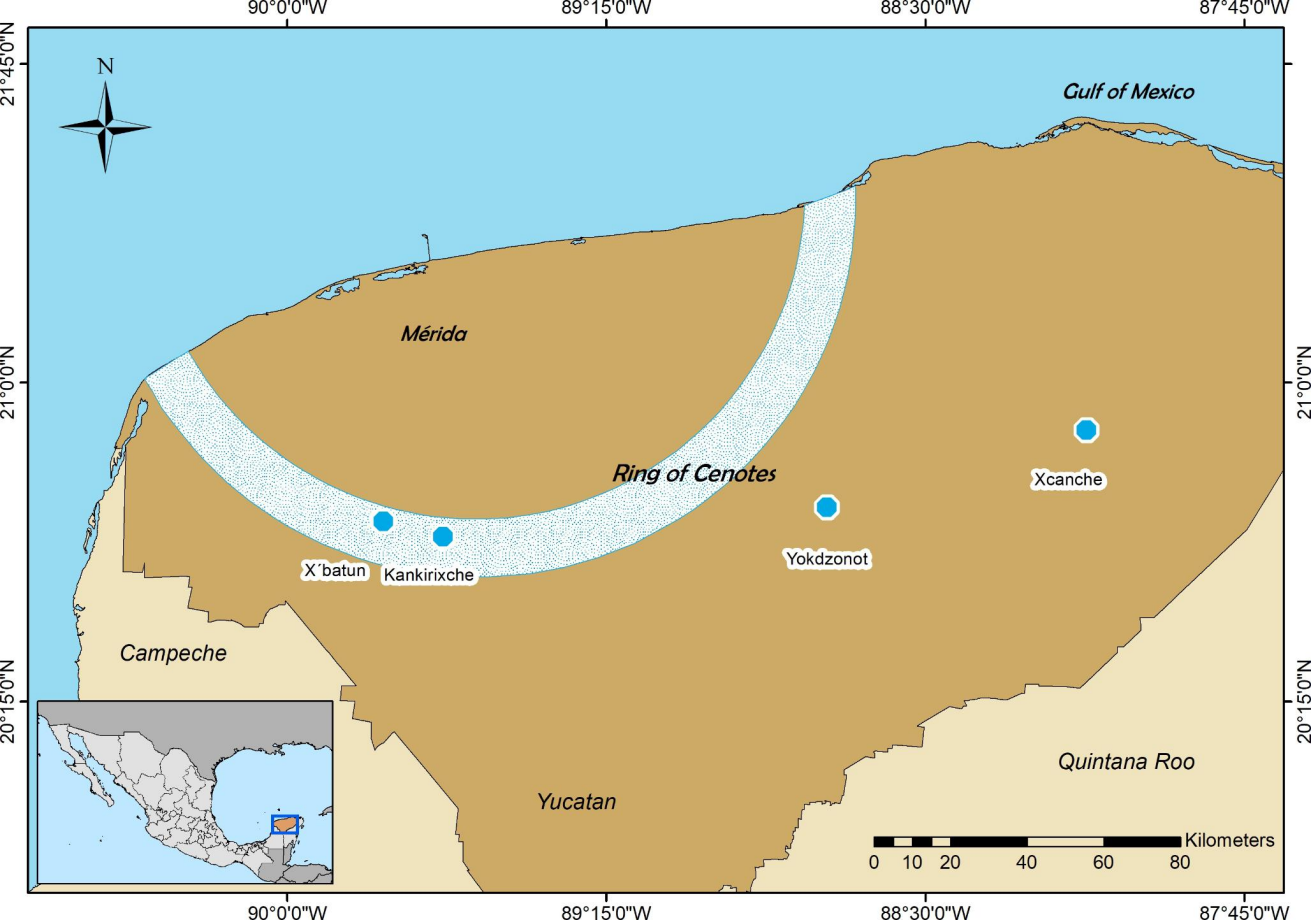
Map showing the location of the study area, the four cenotes sampled, the Ring of Cenotes area, and the limits of the State of Yucatan

The Ring of Cenotes (RC) is in the Yucatan Peninsula’s (YP) northwest region, between parallels 88.5–90.5° W and meridians 20.5–21.5° N (Pérez-Ceballos et al. 2021). A subhumid tropical climate characterizes this region, with temperatures ranging from 25 to 35 °C and annual cumulative rainfall from 555 to 1500 mm y^− 1^ (Gonneea et al. 2014). Within this region, the RC extends from the southwest in Celestun to Dzilam in the northeast, forming a large arc across the middle of the state of Yucatan (Arcega-Cabrera et al. 2014). It is characterized by a complex hydrological system with high permeability and porosity, which makes it vulnerable to contamination (Derrien et al. 2015). Two tourist cenotes along the RC and two to the southeast of the RC were selected for this study (Fig. 1): X’batun, an open-type cenote with a natural slope of approximately 20 m (it is known for the surrounding vegetation that offers shelter for various species); X’Canche, a semi-open type of free fall cenote with a water lens 40 m long by 40 m wide, and a maximum depth of 30 m; Kankirixche, a semi-open type of free fall cenote, that measures 25 m long and 15 m wide, with a depth from 2 to 50 m; and Yokdzonot, an open-type of free fall cenote with a water lens that is 40 m long by 40 m wide and a maximum depth of 45 m (SDS 2021). All these cenotes are used for ecotourism and have ladders and platforms to access the water.

Samples were collected twice. The first collection, a single sampling operation to establish a reference baseline for conditions during the pre-tourists’ season (PH), was conducted two weeks before Easter vacation 2019, in the absence of visitors. The second collection was carried out during Holy Week (HW), as follows: X’Batun and Kankirixche were sampled on April 19th, and Yokdozonot and X’Canche on April 20th. This second sampling campaign was designed to obtain an 8-hour time series (with grab samples every two hours starting at 8 am and finishing at 6 pm, resulting in a total of 6 samples from each cenote, while counting the number of visitors) to assess the potential correlation between water quality variables and touristic activity. The samples from the 2-hour sampling intervals were analyzed separately to obtain an average value for the parameters.

Physicochemical parameters were analyzed in situ using a YSI Professional Plus multiparametric probe (temperature, dissolved oxygen, pH, TDS, ORP, and conductivity) previously calibrated according to the manufacturer’s guidelines. The grab samples of water were collected in the central zone of the water lens’ occupation zone (at approximately 0.5 m below the surface) for nutrients and metals. One liter of water for metals analyses was placed in previously washed high-density polypropylene bottles with HNO_3_. For nutrient analysis, 1 L of water was collected in amber glass bottles, which had been acid cleaned and rinsed with deionized water prior to use. Nitrate (NO_3_^−^), nitrite (NO_2_^−^), ammonium (NH_4_^+^), and orthophosphate (PO_4_^=^) were analyzed by microplate with the methods described in Ringuet et al. (2011). To validate these data, Simple Nutrients in Seawater-whole volume from Sigma-Aldrich (QC3179-500ML, Lot LRAA9336) was used as certified reference material. Water for fecal and total coliforms was sampled in 250 mL sterile polyethylene bags and then analyzed by the membrane filtration method, according to the Mexican Standard NMX-AA-102-SCFI-2006, following the regulations from the Mexican legislation for water destined for human use and consumption (NOM-127-SSA1-1994), which admits reported values both in CFU 100 mL^− 1^ and MPN 100 mL^− 1^. Hardness, sulfates, and chlorides quantification were sampled in 1 L polyethylene bottle, then analyzed and interpreted according to Mexican standards NMX-AA-072-SCFI-2001, NMX-AA-074-SCFI-2014, and NMX-AA-073-SCFI-2001, respectively. For metals analysis 2 mL of HNO_3_ were added to the sample to prevent precipitation or adsorption. Then, the concentration of metals was analyzed using an atomic absorption spectrophotometer (Perkin Elmer Analyst 800) coupled with the reading of a deionized water blank (Arcega-Cabrera and Fargher 2016).

Spearman Rho was calculated to evaluate correlations, using the data from the 2-hour sampling interval, between water quality descriptive variables and the cumulative number of visitors. Data analyses were performed using OriginPro 2019b v9.6.5.169 (OriginLab Corporation).

For multivariate analysis, permutational MANOVA (PERMANOVA) and Principal Coordinate Analysis (PCO) were performed using Primer v 7.0 + PERMANOVA. Data were transformed using the function log10(x + 1) and normalized. The resemblance was calculated using the Euclidean distance of samples (Legendre and Legendre 1998); PERMANOVA was conducted using the permutation of the residuals under a reduced model with 9999 permutations to generate the pseudo-F (Anderson et al., 2001). Permutational multiple pair-wise tests with Monte Carlo test were used to compare the centroids of the combination of two factors, the site (cenotes, four levels) and the condition (PH, HW, two levels).

## Results and Discussion

Changes in water quality parameters, monitored before (PH) and during holy week (HW), show clear variation (Table 1).

**Table 1:** Values for the physical, chemical, and bacteriological water parameters from the sampled cenotes, two weeks before (PH) and during Holy Week (HW) 2019

		Cenote												
Parameter	LOD	X’batun			X’Canche			Kankirixche			Yokdzonot			
		^PH (BP)^	^HW^	^SD^	^PH(BP)^	^HW^	^SD^	^PH(BP)^	^HW^	^SD^		^PH(BP)^	^HW^	^SD^
Temperature (°C)		27.4	**27.7**	0.08	25.3	**25.7**	0.16	27.4	27.4	0.04		25.2	25.4	0.05
pH		6.73	**6.91**	0.02	6.91	**7.08**	0.01	7.01	6.92	0.07		7.49	7.37	0.06
O_2_ (mg L^− 1^)		3.52	**4.07**	0.94	3.71	**4.91**	0.64	3.35	2.91	0.36		4.12	3.72	0.39
TDS (mg L^− 1^)		1440	1190	15.1	715	585	0.01	1220	1140	0.01		676	637	0.01
Conductivity (µS cm^− 1^)		2320	1920	22.9	902	**912**	3.43	1960	1830	2.23		955	**987**	2.25
ORP (mV)		-6.10	-7.58	12.4	-49.6	**32.5**	19.1	-29.6	-45.5	4.27		-45.8	-60.5	4.43
NO_3_^−^ (mg L^− 1^)	0.01	ND	ND	--	0.04	**7.52**	3.08	ND	**0.02**	0.01		ND	**0.05**	0.03
NO_2_^−^ (mg L^− 1^)	0.02	ND	**0.03**	0.02	0.05	ND	--	ND	ND	--		0.06	**0.06**	0.02
NH_4_^+^ (mg L^− 1^)	0.04	ND	**0.09**	0.06	0.04	**0.05**	0.06	ND	**0.01**	0.01		0.32	0.10	0.06
PO_4_^3−^ (mg L^− 1^)	0.01	ND	ND	--	ND	ND	--	ND	ND	--		0.02	ND	--
SiO_4_^4−^(mg L^− 1^)	0.05	4.52	4.07	0.72	3.18	2.28	0.37	3.66	**4.05**	0.49		3.70	3.59	0.23
Mg (mg L^− 1^)	1.2E-03	58.7	50.9	18.4	6.80	**16.6**	4.77	62.6	43.1	10.8		22.6	**28.3**	6.49
Na (mg L^− 1^)	6.0E-03	223	156	143	61.0	**77.7**	61.2	205	103	67.4		57.0	**144**	106
Ca (mg L^− 1^)	5.0E-04	495	**674**	45.9	585	452	84.7	523	**624**	83.4		497	488	40.4
K (mg L^− 1^)	4.0E-04	9.79	**10.4**	0.17	5.41	**5.80**	0.33	8.54	**8.81**	0.21		6.86	7.32	0.16
Sr (mg L^− 1^)	7.5E-03	3.39	3.22	0.02	0.52	**0.63**	0.02	2.88	**3.16**	0.33		0.74	**0.90**	0.05
Zn (mg L^− 1^)	1.7E-03	0.05	0.01	0.01	0.01	ND	--	0.01	0.01	0.01		ND	0.01	--
Alkalinity (mg L^− 1^ CaCO_3_)		294	**295**	2.45	319	319	1.75	296	293	0.62		261	258	2.38
Chlorides (mg L^− 1^ Cl^−^)		577	574	39.9	272	**290**	54.8	398	517	102		259	**332**	75.7
Sulphates (mg L^− 1^ SO_4_^=^)		195	**282**	44.9	26.4	**32.2**	23.1	142	**193**	28.8		38.6	**40.2**	24.0
Cu (µg L^− 1^)	4.0E-04	14	1.07	0.45	5.61	2.72	1.78	ND	**1.66**	0.95		6.06	2.14	2.01
Cr (µg L^− 1^)	0.01	1.22	ND	--	3.20	1.44	1.79	ND	**5.41**	1.17		3.12	**12.7**	0.65
Cd (µg L^− 1^)	4.8E-03	0.14	**0.25**	--	0.06	**0.71**	0.08	0.02	ND	--		0.06	**0.19**	0.26
As (µg L^− 1^)	1.3E-03	0.59	0.09	0.01	0.04	0.04	0.03	0.65	0.08	0.01		0.45	0.10	0.01
Fecal coliforms (CFU 100 mL^− 1^)		50	**439**	309	40	**52**	23.6	174	**318**	282		118	38	15.5
Total coliforms (CFU 100 mL^− 1^)		uncountable	**960**	421	158	**1270**	586	185	**1320**	1780		uncountable	**421**	232
*Enterococcus* (CFU 100 mL^− 1^)		40	**87**	59.9	21	**32**	26.7	uncountable	252	104		uncountable	133	62.4

PH(BP): pre-holiday season (baseline). HW: Holy Week 2019, mean values for the 6 samples from each cenote. ND: Not Detected.

SD: Standard deviation for HW values. LOD: Limit of detection.

Values in bold represent an increment from previous data.

**Fig. 2:**
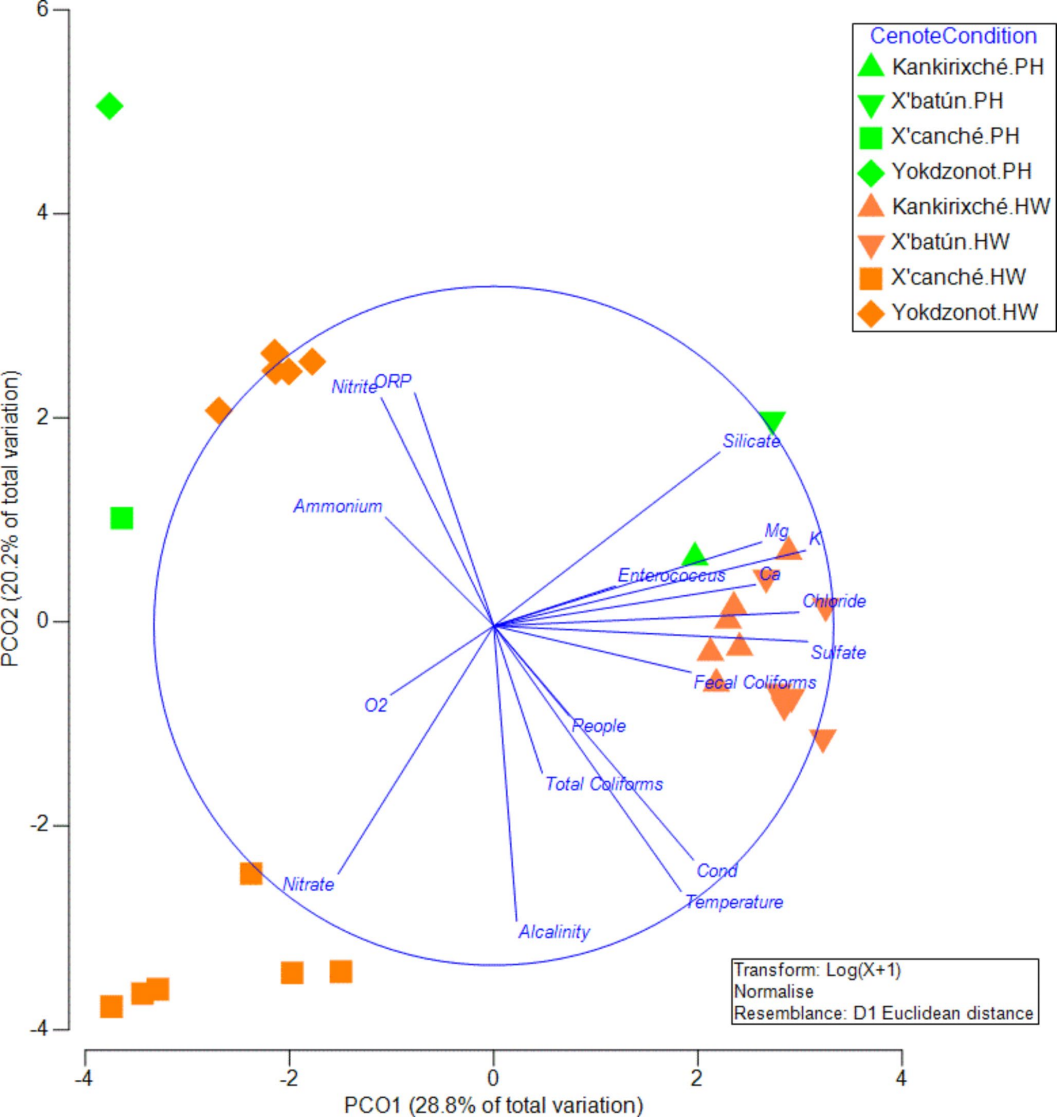
Principal Coordinate Analysis (PCO) of water quality variables two weeks before (PH, green) and during Holy Week 2019 (HW, orange) for cenotes Kankirixché, X’batun, X’canché, and Yokdzonot. All the variables listed in the methods were used for the analysis, but only those with correlations higher than 0.5 are depicted

PCO applied to water quality parameters explained 49.02% of the total variation in the first two principal coordinates (Fig. 2). Also, PERMANOVA indicated a significant interaction between the factors “cenote” and “condition” (Pseudo-F = 3.77, p = 0.0001, 9921 permutations), indicating that the water quality parameters varied differently in the cenotes. Furthermore, *post hoc* paired comparisons indicated significant differences between the conditions before (PH) and during vacation (HW) for Kankirixche (p = 0.022), X’batun (p = 0.009), X’Canche (p = 0.032) and Yokdzonot (p = 0.01) although these significant differences could be the result of touristic use, there is still an uncertainty related with the environmental hydrogeochemistry and location of each cenote that should be kept in mind.

The indicators that contributed most to the PCO variation in the first principal coordinate (horizontal) were Sr (0.97), K (0.91), sulfates (0.94), and chlorides (0.89). The indicators with the highest contribution to the second coordinate (vertical) were alkalinity (-0.86), temperature (-0.78), conductivity (-0.69), nitrate (-0.73) and ORP (0.69). The ordination showed three groups. The first group includes samples from the Yokdzonot cenote, which showed the highest reducing condition (ORP), as well as high ammonia, and nitrites. These conditions may be present in this cenote because of its depth, as well as the significant amounts of degrading organic matter at the bottom could promote reducing conditions in the water column (Schmitter-Soto et al. 2002).

The second group is formed by the X’batún and Kankirixche cenotes. It is characterized by higher concentrations of silicate, Ca, Sr, Mg, chlorides, and sulfates. This could be a result of location, since in that region sulfates, sodium, and chlorides increase towards the coast (Pérez-Ceballos 2021). Also, these cenotes were the ones with the highest concentration of bacteria, especially fecal coliforms. This could be expected, because these cenotes were the ones that had the highest number of tourists (Table 2).

Following the classification by Schmitter-Soto et al. (2002), nutrient concentration in lotic cenotes is expected to be lower than in lentic ones, because of the difference in turnover rate. Group three includes the X’Canche cenote. It showed the highest concentration of NO_3_^−^ (7.52 mg L^− 1^), yet all the measured concentrations were below the Mexican guideline values (9.9 mg L^− 1^ of NO_3_^−^ - NOM-127-SSA1-1994). Historically, nitrate concentrations in the groundwater of the state of Yucatan have ranged between 15.51 and 70.61 mg L^− 1^ for Merida (Fabro et al. 2015) and 0.6 and 17.7 mg L^− 1^ for the ring of cenotes area (Pérez-Ceballos et al. 2021); these high concentrations have been linked to human activities (Long et al. 2018). Nevertheless, the opposite was documented for X’Batun, Kankiriche and Yokdzonot, since sampling was conducted during the dry season. During this season, in absence of runoff, organic matter inputs are limited, and the type of nitrogenated species (nitrates, nitrites, or ammonia) will depend on the redox potential of the water (Pérez-Ceballos et al. 2012). Therefore, nitrates increase in the X’Canche cenote (present as nitrates as a result of the OD and ORP) during the dry season could be related to human activities, as has been previously reported for other sinkholes (Arcega-Cabrera et al. 2021). Human discharges from inland and littoral activities in Yucatan, generate around 2% of the nitrogen (Aranda-Cirerol et al. 2010). Therefore, tourism should not be underestimated as a nutrient source since sudden increases in nutrient concentrations at a specific site, resulting from crowding people during a comparatively short period of time (10 h), may not be buffered by the ecosystem. This could promote an overload of nutrients in time with consequences such as the sudden growth of opportunistic species.

Regarding fecal contamination, since there is no specific legislation for inland recreational waters in Mexico, we use the values for fecal coliform previously established in the US EPA’s 1986 recreational waters criteria (US EPA 2012). According to this fecal coliform standard (200 CFU 100 mL^− 1^), Kankirixche and X’Batun exceeded the limit during Holy Week. This could be a result of the sudden increase in visitors (Table 2), bathing in a smaller water lens area, since these cenotes have the most restricted surface area. In Fig. 2, it is possible to note the increase of all the microbiological parameters during HW, and also that the number of tourists augments the presence of bacteria.

**Table 2:** Cumulative number of visitors at the time of sampling in the cenotes during Holy Week 2019

	Cenote			
Clock Time	X’Batun	X’Canche	Kankirixche	Yokdzonot
8:00	0	0	0	0
10:00	28	17	6	2
12:00	172	45	93	17
14:00	306	142	220	122
16:00	400	232	319	243
18:00	454	256	370	282

The presence of *Enterococcus* in X’Batun and Kankirixche (with the smallest water lens diameters of 20 and 25 m, respectively), which is comparatively more resilient, may indicate leaks from septic tanks or even inputs from other anthropogenic activities in the area (Pérez-Ceballos 2012, Arcega-Cabrera et al. 2014, 2021). Variables showing a moderate to strong correlation with the total number of visitors in the sampled cenotes are shown in Table 3.

**Table 3:** Correlation (r) of tourist affluence with water quality parameters in the cenotes

	Cenote			
Parameter	X’Batun	X’Canche	Kankirixche	Yokdzonot
	r value			
pH	**0.794**	0.213	0.086	-0.667
O_2_ (mg L^− 1^)	**0.714**	0.371	0.314	**0.657**
TDS (mg L^− 1^)	**0.845**	--	--	--
NO_3_^−^ (mg L^− 1^) nitrate	-0.943	--	**0.600**	0.500
NH_4_^+^ (mg L^− 1^) ammonium	0.086	0.086	**0.698**	**0.700**
Cu (µg L^− 1^)	0.371	0.314	0.314	-0.100
Cr (µg L^− 1^)	--	0.499	0.580	0.584
Fecal coliforms (CFU 100 mL^− 1^)	0.543	-0.543	**0.886**	0.435
Total coliforms (CFU 100 mL^− 1^)	0.428	0.257	**0.600**	**0.829**
*Enterococcus* (CFU 100 mL^− 1^)	**0.600**	-0.429	**0.943**	0.543

Values in bold represent a positive correlation between the water quality parameter and the number of visitors.

The number of significant correlations to the total number of visitors varies among sites; this could be the result of differences in water lens area, depth, water residence time, cenote shape, etc. Number of visitors is moderate to strongly correlated with at least one of the fecal matter indicators in three cenotes. Results show that a higher number of visitors promoted a higher concentration of coliforms and/or *Enterococcus*, sometimes surpassing normative values. This outcome may have a negative effect on the cenote and on tourists’ health (Almeida et al. 2007).

## Conclusions

Monitoring programs in cenotes play a significant role in a sustainable use, promoting good water quality, and safe recreational use. The use of cenotes for water-based tourism purposes is increasing in Yucatan. However, even though their use for recreational activities can be beneficial for an individual’s health, it can also be damaging if the water is contaminated and eventually becomes unsafe. Our findings concerning the impact of visitors on water quality provide helpful information for informing decision-making and the creation of stronger regulations for the protection of karstic cenotes in Yucatan. These include; i) specifications for infrastructure nearby and in the cenotes, ii) regular monitoring of water quality, iii) improvement of wastewater treatment systems in nearby urban areas, and, iv) prevention rules for tourists visiting cenotes, among others. Also, in situ management programs according to a specific cenote’s characteristics and its tourist use are strongly recommended.

## References

[CR1] Almeida CA, Quintar S, González P, Mallea MA (2007). Influence of urbanization and tourist activities on the water quality of the Potrero de los Funes River (San Luis - Argentina). Environ Monit Assess.

[CR2] Anderson MJ (2001). A new method for non-parametric multivariate analysis of variance. Austral Ecol.

[CR3] Angyal D, Chávez-Solís EM, Liévano-Beltrán LA, Magaña B, Simoes N, Mascaró M (2020). New distribution records of subterranean crustaceans from cenotes in yucatan (Mexico). ZooKeys.

[CR4] Aranda-Cirerol N, Comín F, Herrera-Silveira J (2011). Nitrogen and phosphorus budgets for the Yucatán littoral: an approach for groundwater management. Environ Monit Assess.

[CR6] Arcega-Cabrera F, Fargher LF (2016). Education, fish consumption, well water, chicken coops, and cooking fires: using biogeochemistry and ethnography to study exposure of children from Yucatan, Mexico to metals and arsenic. Sci Total Environ.

[CR5] Arcega-Cabrera F, Velázquez-Tavera N, Fargher L, Derrien M, Noreña-Barroso E (2014). Fecal sterols, seasonal variability, and probable sources along the ring of cenotes, Yucatan, Mexico. J Contam Hydrol.

[CR8] Arcega-Cabrera F, Sickman J, Fargher L, Herrera-Silveira J, Lucero D, Oceguera-Vargas I, Lamas-Cosío E, Robledo-Ardila P (2021) Groundwater Quality in the Yucatan Peninsula: insights from stable isotope and Metals Analysis. Groundwater 1–14. 10.1111/gwat.13109 10.1111/gwat.13109 33948945

[CR9] Arshad H, Mehmood MZ, Shah MH, Abbasi AM (2020). Evaluation of heavy metals in cosmetic products and their health risk assessment. Saudi Pharm J.

[CR10] Ayenimo JG, Yusuf AM, Adekunle AS, Makinde OW (2010). Heavy metal exposure from personal care products. Bull Environ Contam Toxicol.

[CR11] Bauer-Gottwein P, Gondwe BRN, CharvetG, Marín LE, Rebolledo-Vieyra M, Merediz-Alonso G (2011). Review: the Yucatán Peninsula karst aquifer, Mexico. Hydrogeol J.

[CR12] Borbolla-Vazquez J, Ugalde-Silva P, León-Borges J, Díaz-Hernández JA (2020). Total and faecal coliforms presence in cenotes of Cancun. Quintana Roo Mexico BioRisk.

[CR13] Camacho-Cruz KA, Ortiz-Hernández MC, Sánchez A, Carrillo L, De Jesús Navarrete A (2020). Water quality in the eastern karst region of the Yucatan Peninsula: nutrients and stable nitrogen isotopes in turtle grass, Thalassia testudinum. Environ Sci Pollut Res.

[CR14] Casas-Beltran DA, Hernández-Pedraza M, Alvarado-Flores J (2021). Estimation of the discharge of sunscreens in aquatic environments of the mexican caribbean. Environ - MDPI.

[CR15] Cejudo E, Acosta-González G, Ortega-Camacho D, Tun-Rosado GE (2020) Changes in the hydrochemistry of a karstic lake in Yucatan, Mexico. Environ Earth Sci 79(5). 10.1007/s12665-020-8838-3

[CR16] Derrien M, Cabrera FA, Tavera NLV, Kantún Manzano CA, Vizcaino SC (2015). Sources and distribution of organic matter along the Ring of Cenotes, Yucatan, Mexico: sterol markers and statistical approaches. Sci Total Environ.

[CR18] Enseñat-Soberanis F, Blanco-Gregory R, Mondragón-Mejía J, Simoes N, Moreno-Acevedo E, Ortega I (2020). Crowding standards and willingness to pay at cenotes (sinkholes) of the Yucatan Peninsula: a comparative analysis of local, national and international visitors. J Ecotourism.

[CR19] Fabro-Rojas AY, Pacheco-Ávila JG, Eteller-Alberich MV, Cabrera-Sansores SA, Camargo-Valero MA (2015). Spatial distribution of nitrate health risk associated with groundwater use as drinking water in Mérida, México. Appl Geogr.

[CR20] Gonneea ME, Charette MA, Liu Q, Herrera-Silveira JA, Morales-Ojeda SM (2014). Trace element geochemistry of groundwater in a karst subterranean estuary (Yucatan Peninsula, Mexico). Geochim Cosmochim Acta.

[CR21] Hernández-Mendoza H, Piña Leyte-Vidal JJ, Romero-Guzmán ET, Rios-Lugo MJ, Medellín-Castillo NA (2022) Relationship of thorium, uranium isotopes and uranium isotopic ratios with physicochemical parameters in cenote water from the Yucatán Peninsula. Appl Radiat Isot September 110470. 10.1016/j.apradiso.2022.110470 10.1016/j.apradiso.2022.110470 36209646

[CR22] Herrera-Silveira JA, Comin FA, Aranda-Cirerol N, Troccoli L, Capurro L (2004). Coastal water quality assessment in the Yucatan Peninsula: management implications. Ocean Coast Manag.

[CR23] Hoogesteijn Reul ALH, Febles-patrón JL, Nava-galindo VA (2015). La contaminación fecal en cenotes de interés turístico y recreacional del estado de Yucatán. Ingeniería.

[CR24] Leal-Bautista RM, Hernández-Zárate GM, Jaime MNA, Cuevas RG, Velázquez Oliman G (2011). Pathogens and pharmaceuticals pollutants as indicators of contamination at the Northeasthern Aquifer of Quintana Roo. Trop Subtrop Agroecosystems.

[CR25] Lebedeva EV, Mikhalev DV, Nekrasova LA (2017). Evolutionary stages of the karst-anthropogenic system of the Yucatán Peninsula. Geogr Nat Resour.

[CR26] Legendre P, Legendre L (1998). Numerical ecology.

[CR27] León-Borges JA, Viveros-Jiménez F, Rodríguez-Mata AE, Lizardi-Jiménez MA (2020). Hydrocarbon contamination patterns in the Cenotes of the mexican Caribbean: the application of principal component analysis. Bull Environ Contam Toxicol.

[CR28] Long DT, Pearson AL, Voice TC, Polanco-Rodríguez AG, Sanchez-Rodríguez EC, Xagoraraki I, Concha-Valdez FG, Puc-Franco M, Lopez-Cetz R, Rzotkiewicz AT (2018). Influence of rainy season and land use on drinking water quality in a karst landscape, state of Yucatán. Mexico Appl Geochem.

[CR29] Melo Zurita MdeL (2019). Holes, subterranean exploration and affect in the Yucatan Peninsula. Emot Space Soc.

[CR30] Nava-Galindo VA (2015) Percepción, conocimiento local y descripción de la calidad del agua de cenotes de interés turístico y recreacional. Master´s thesis. Centro de Investigación y Estudios Avanzados del Instituto Politécnico Nacional (CINVESTAV-IPN), Yucatán

[CR32] NMX-AA-072-SCFI-2001 Análisis de agua, determinación de dureza total en aguas naturales, residuales y residuales tratadas, método de prueba. Available at https://www.gob.mx/cms/uploads/attachment/file/166788/NMX-AA-072-SCFI-2001.pdf

[CR33] NMX-AA-073-SCFI-2001 Análisis de agua, determinación de cloruros totales en aguas naturales, residuales y residuales tratadas, método de prueba. Available at https://www.gob.mx/cms/uploads/attachment/file/166789/NMX-AA-073-SCFI-2001.pdf

[CR34] NMX-AA-074-SCFI-2014 Análisis de agua, medición del ion sulfato en aguas naturales, residuals y residuales tratadas, método de prueba. Available at https://www.gob.mx/cms/uploads/attachment/file/166149/nmx-aa-074-scfi-2014.pdf

[CR31] NMX-AA-102-SCFI -2006 Calidad de agua-detección y enumeración de organismos coliformes, organismos coliformes termotolerantes y Escherichia coli presuntiva, método de filtración en membrana. Available at https://www.gob.mx/cms/uploads/attachment/file/166804/NMX-AA-102-SCFI-2006.pdf

[CR35] NOM-127-SSA 1-1994 Salud Ambiental, agua para uso y consume humano, límites permisibles de calidad y tratamientos a que debe someterse el agua para su potabilización. Available at https://www.pediatria.gob.mx/archivos/burbuja/13.4_NOM-127-SSA1-1994_Salud_Ambiental_Agua_limites_permisibles_de_calidad.pdf

[CR36] Onac BP, van Beynen P (2021) Caves and Karst. In: Alderton D, Scott A, Elias (eds) Encyclopedia of Geology, 2nd edn. Academic Press, pp 495–509. 10.1016/B978-0-12-409548-9.12437-6

[CR37] Pérez-Ceballos R, Pacheco-Ávila J, Hernández-Arana (2012). Regionalization based on water chemistry and physicochemical traits in the ring of cenotes, Yucatan, Mexico. J Cave Karst Stud Natl Speleological Soc Bull.

[CR38] Pérez-Ceballos R, Canul-Macario C, Pacheco-Castro R, Pacheco-Ávila J, Euán-Ávila J, Merino-Ibarra M (2021) Regional hydrogeochemical evolution of groundwater in the ring of cenotes, Yucatán (Mexico): an inverse modelling approach. Water (Switzerland) 13(5). 10.3390/w13050614

[CR40] Ringuet S, Sassano L, Johnson ZI (2011). A suite of microplate reader-based colorimetric methods to quantify ammonium, nitrate, orthophosphate and silicate concentrations for aquatic nutrient monitoring. J Environ Monit.

[CR42] Schmitter-Soto JJ, Comín FA, Escobar-Briones E, Herrera-Silveira J, Alcocer J, Suárez-Morales E, Elías-Gutiérrez M, Díaz-Arce V, Marín LE, Steinich B (2002). Hydrogeochemical and biological characteristics of cenotes in the Yucatan Peninsula (SE Mexico). Hydrobiologia.

[CR43] Scholz T, Vargas-Vázquez J, Moravec F, Vivas-Rodríguez C, Mendoza-Franco E (1995). Cenotes (sinkholes) of the Yucatan Peninsula, Mexico, as a habitat of adult trematodes of fish. Folia Parasitol.

[CR44] SDS (2021) Cenotes Turísticos de Yucatán. Secretaría de Desarrollo Sustentable, Gobierno del Estado de Yucatán https://sds.yucatan.gob.mx/cenotes-grutas/cenotes-turisticos-de-yucatan.php Accessed 26 March 2021

[CR45] SEFOTUR (2021) Listado de Cenotes Turístucos 2021 del Estado de Yucatán. Secretaría de Fomento Turístico del Gobierno del Estado de Yucatán, Gobierno del Estado de Yucatán http://www.sefotur.yucatan.gob.mx/files-content/general/fa1702c19901227a147be57ee0070f02.pdf Accessed 26 March 2021

[CR41] Silva KB, Mattos JB (2020). A spatial approach for the management of groundwater quality in tourist destinations. Tour Manag.

[CR46] US EPA (2012) Recreational Water Quality Criteria. Office of Water 820-F-12-058

[CR47] Visser AN, Lehmann MF, Rügner H, D’Affonseca FM, Grathwohl P, Blackwell N, Kappler AA, Osenbrück K (2021). Fate of nitrate during groundwater recharge in a fractured karst aquifer. Hydrogeol J.

[CR48] Yang P, Wang Y, Wu X, Chang L, Ham B, Song L, Groves C (2020). Nitrate sources and biogeochemical processes in karst underground rivers impacted by different anthropogenic input characteristics. Environ Pollut.

